# Salt-Tolerant Halophyte Rhizosphere Bacteria Stimulate Growth of Alfalfa in Salty Soil

**DOI:** 10.3389/fmicb.2019.01849

**Published:** 2019-08-14

**Authors:** Jennifer Kearl, Caitlyn McNary, J. Scott Lowman, Chuansheng Mei, Zachary T. Aanderud, Steven T. Smith, Jason West, Emily Colton, Michelle Hamson, Brent L. Nielsen

**Affiliations:** ^1^Department of Microbiology and Molecular Biology, Brigham Young University, Provo, UT, United States; ^2^The Plant Endophyte Research Center, The Institute for Advanced Learning and Research, Danville, VA, United States; ^3^Department of Plant and Wildlife Sciences, Brigham Young University, Provo, UT, United States

**Keywords:** halophyte, halophilic bacteria, rhizosphere, endophyte, microbiome, salt tolerance, plant growth-promoting rhizobacteria

## Abstract

Halophytes are plants that are adapted to grow in saline soils, and have been widely studied for their physiological and molecular characteristics, but little is known about their associated microbiomes. Bacteria were isolated from the rhizosphere and as root endophytes of *Salicornia rubra, Sarcocornia utahensis,* and *Allenrolfea occidentalis*, three native Utah halophytes. A total of 41 independent isolates were identified by 16S rRNA gene sequencing analysis. Isolates were tested for maximum salt tolerance, and some were able to grow in the presence of up to 4 M NaCl. Pigmentation, Gram stain characteristics, optimal temperature for growth, and biofilm formation of each isolate aided in species identification. Some variation in the bacterial population was observed in samples collected at different times of the year, while most of the genera were present regardless of the sampling time. *Halomonas*, *Bacillus*, and *Kushneria* species were consistently isolated both from the soil and as endophytes from roots of all three plant species at all collection times. Non-culturable bacterial species were analyzed by Illumina DNA sequencing. The most commonly identified bacteria were from several phyla commonly found in soil or extreme environments: Acidobacteria, Actinobacteria, Bacteroidetes, Chloroflexi, and Gamma- and Delta-Proteobacteria. Isolates were tested for the ability to stimulate growth of alfalfa under saline conditions. This screening led to the identification of one *Halomonas* and one *Bacillus* isolate that, when used to inoculate young alfalfa seedlings, stimulate plant growth in the presence of 1% NaCl, a level that significantly inhibits growth of uninoculated plants. The same bacteria used in the inoculation were recovered from surface sterilized alfalfa roots, indicating the ability of the inoculum to become established as an endophyte. The results with these isolates have exciting promise for enhancing the growth of inoculated alfalfa in salty soil.

## Introduction

Many agricultural areas in the southwestern United States and other parts of the world rely heavily on irrigation. In many of these areas, soil salinity has been increasing due to drought combined with poor irrigation practices. Most crop plants are sensitive to salt, which leads to reductions in production (reviewed in Gul et al., 2014). The severity of increasing soil salinity will likely intensify with growing food demand and degradation and loss of prime agricultural land. According to the USDA salinity laboratory website^1^, about 15% of cultivated land globally is irrigated, but irrigated areas account for up to 40% of the total food harvest. In the U.S., salinity of soil and water affects about 30% of all irrigated land, while about 50% of irrigated land worldwide is affected. Salinity increases in irrigated areas due to soluble salts carried in the irrigation water that remain in the soil after evaporation and transpiration. Unless these salts are leached from the soil, they accumulate to levels that are inhibitory to plant growth and may lead to soils becoming sodic, causing degradation of soil structure to affect water and root penetration along with other problems (Gul et al., 2014). According to USDA estimates, about 10 million hectares are lost globally each year as a result of salinity and/or waterlogging. Salinity and other environmental stresses will require new approaches to maintain an adequate food supply. The potential of rhizobacteria to stimulate plant growth in poor quality soil is an important component in addressing this problem.

Halophytes are naturally salt-tolerant plants that have evolved to grow in saline soils; different halophyte species have different salt tolerance levels (Flowers and Colmer, 2015). Much of the state of Utah is a high desert with saline soils, and a wide variety of halophytes are native to this area. In the study area, we have focused on primarily three halophyte species: *Salicornia rubra*, *Sarcocornia utahensis*, and *Allenrolfea occidentalis* (Weber, 2016). Over the past few decades, considerable progress has been made in understanding physiological mechanisms and gene expression changes involved in salt tolerance in halophytes (Shabala, 2013; Diray-Arce et al., 2015). Some halophytes have been developed or have potential for use as crop plants (Khan et al., 2009; Gul et al., 2014). However, little is known about the potential contribution of microorganisms associated with these plants in the soil, on plant surfaces, or within plant tissues. Microbes found in the rhizosphere (rhizobacteria) or within plant tissues including roots (endophytes) have the potential to contribute significantly to the ability of plants to adapt to adverse conditions (Numan et al., 2018). Characterizing soil bacteria from saline environments may lead to identification of beneficial microorganisms for use as inoculants to stimulate growth of non-host plants under saline conditions.

There is a growing number of publications on plant growth promotion by salt-tolerant (halophilic) rhizobacteria isolated from halophyte species in saline soils (Mapelli et al., 2013; Rajput et al., 2013; Ruppel et al., 2013; Li et al., 2016; Orhan, 2016; Sharma et al., 2016; Kataoka et al., 2017; Palacio-Rodriguez et al., 2017; reviewed in Etesami and Beattie, 2018; Numan et al., 2018). Each of these publications identify different microbial species associated with a range of plant and growing environments, suggesting that there may be microbe-host specific interactions. These reports provide evidence to show that halophiles isolated from the rhizosphere or as endophytes of halophytes may be used as inocula to stimulate growth of salt-sensitive crops. In addition to bacteria in the rhizosphere, some endophytes are capable of stimulating plant growth under saline conditions, including species of S*phingomonas, Bacillus, Enterobacter,* and *Pantoea*, which enhance salt tolerance of hybrid elephant grass (Li et al., 2016).

Little previous work has been published on microbiomes associated with native halophytes in desert areas of the United States. Halophilic microbes in and near the Great Salt Lake and other marine environments have been studied, but aquatic species are different from those found in desert soil. We have focused on the microbiomes of three halophyte species that grow in a highly saline area south of Utah Lake where soil salinity is between 16 and 100 dS/m (compared to local land where alfalfa is growing that is 0.7–1.6 dS/m and ocean water, which is about 55 dS/m). DNA sequence analysis of the isolates identified species of a number of known halophilic genera. Some isolates are capable of growth in up to 4 M NaCl, and two isolates show promise for use as inocula for alfalfa to stimulate growth in salty soil.

## Materials and Methods

### Collection of Samples

**Figure 1:**
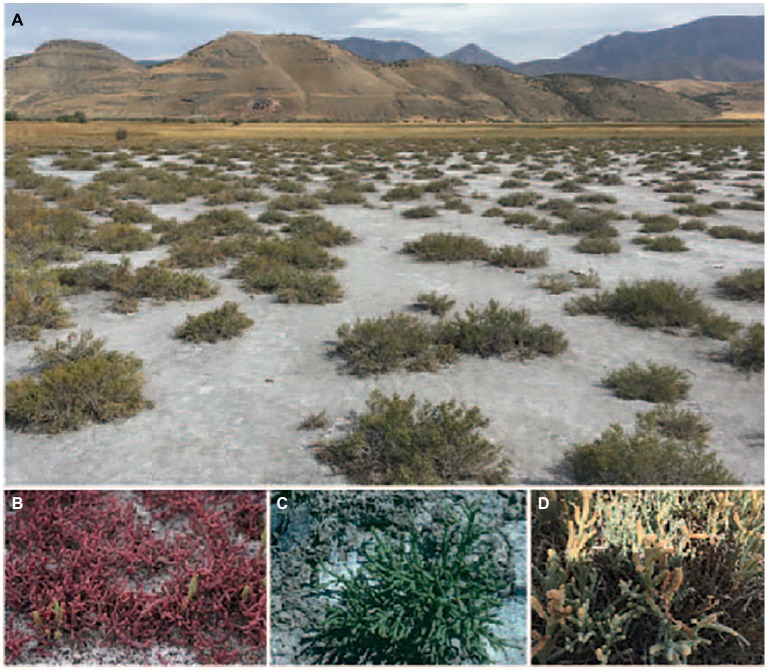
Collection site south of Utah Lake near Goshen, Utah. **(A)** shows an overall view of the site. **(B–D)** are close-up photos of each of the three halophyte species: **(B)** *Salicornia rubra*; **(C)** *Sarcocornia utahensis;* **(D)** *Allenrolfea occidentalis.*

During the past 2 years, we have made six collection trips to a study site near Goshen, Utah (coordinates: 39:57:06 N 111:54:03 W, 1360 m above sea level; Figure 1; Gul et al., 2009). At this study site, there are three predominant halophyte species, each native to Utah (*Salicornia rubra*, *Sarcocornia utahensis,* and *Allenrolfea occidentalis*; all are members of the same subfamily, Salicornioideae). Individual plants of each of the three species were removed from the ground, and samples of soil adhering to the roots and root tissue were separately collected into sterile tubes for transport to the lab. Disposable gloves were worn for each sample to avoid cross-contamination between samples and from human-associated microbes. Soil was also collected from bare areas where no plants were growing for comparison. Soil was analyzed by the BYU Soils Lab for salinity level and pH. Soil salinity was measured using a Beckman RC-16C conductivity bridge to measure electrical conductivity as dS/m. Soluble salts and pH were measured in saturated soil pastes. Soil samples were mixed with deionized water, the saturated mix was allowed to sit overnight for the soil to settle, and the pH of the liquid was measured with a standard pH meter.

### Isolation and Characterization of Bacteria

Rhizosphere soil samples were vortexed in buffer [0.5 g sample in 1 ml 1 X PBS (phosphate buffered saline)] and plated on Luria broth (LB) agar plates containing 1 M NaCl. To isolate endophytic bacteria, root samples were surface sterilized (by washing twice in sterile distilled water, once for 10 min in 70% ethanol, and twice in sterile PBS) and ground in PBS buffer. Cultures were re-streaked on LB media containing increasing amounts of NaCl (1 M, 2 M, 3 M, 4 M) to determine maximum salt tolerance of each isolate. Bacterial isolates were also tested for maximum salt tolerance on M9 minimal salts media agar plates. Colony morphology, pigmentation, and the temperature range of growth for each isolate were also determined. Individual colonies were used to inoculate liquid LB + 0.25 M NaCl and incubated overnight with shaking at 30°C. Stock cultures of each isolate were stored at −80°C in 20% glycerol.

### Bacterial Identification

To identify the bacteria, genomic DNA was obtained from individual isolates using a DNA isolation protocol that involves lysis and digestion of nucleases with proteinase K (Chachaty and Saulnier, 2000). For each sample, the 16S ribosomal RNA (rRNA) gene was amplified by PCR using the 8F and 1492R primers (Turner et al., 1999) for sequence determination at the Brigham Young University Sequencing Center (http://dnac.byu.edu/; Sanger sequencing protocol). Sequences obtained were used to identify the genus and species by BLAST search of the NIH/NCBI bacterial database. Forty one individual sequences were submitted to GenBank, accession numbers MK873873-MK873913. Colony morphology and Gram staining were utilized to assist in identification of the species (Vreeland et al., 1980; Zhang et al., 2007).

To identify nonculturable bacteria in the halophyte rhizosphere samples, we characterized bacterial communities on roots using barcoded next-generation sequencing of the 16S rRNA gene in a metagenomic approach. We extracted genomic DNA from 1.0 g of rhizosphere soil using the DNeasy Powersoil Kit (Qiagen Inc., Germantown, MD, USA). The V4 region of the 16S rRNA gene was amplified using the bacterial specific primer set 515F and 806R with unique 12 nt error correcting Golay barcodes (Aanderud et al., 2016). Barcoded samples were purified (Agencourt AMPure XP PCR Purification Beckman Coulter Inc., Brea, CA, USA) and normalized with a SequalPrep Normalization Plate Kit (Invitrogen, Carlsbad, CA, USA); pooled at approximately equimolar concentrations after being quantified with an Agilent 2100 Bioanalyzer (Agilent, Santa Clara, CA, USA). All samples were sequenced at the Brigham Young University DNA Sequencing Center^2^ *via* 2 × 250 bp paired-end sequencing on an illumina HiSeq 2500 System (HiSeq Rapid SBS Kit v2, illumine, San Diego, CA, USA). All sequences were processed using the mothur (v. 1.39.0) pipeline (https://www.mothur.org/wiki/MiSeq_SOP; Schloss et al., 2009; Kozich et al., 2013). After removing barcodes and primers, we eliminated sequences that were < 250 bp in length or sequences possessing homopolymers longer than 8 bp. We then denoised the sequences with AmpliconNoise (Quince et al., 2011), removed chimeras with UCHIME (Edgar et al., 2011), and eliminated chloroplast, mitochondrial, archaeal, and eukaryotic gene sequences based on reference sequences from the Ribosomal Database Project (Cole et al., 2009). Sequences were aligned against the SILVA database (silva.nr_v132; Pruesse et al., 2007) with the SEED aligner to create operational taxonomic units (OTUs) based on uncorrected pairwise distances at 97% sequence similarity. Phylogenetic identity of the OTUs was determined with the SILVA database and all samples were rarified to a common sequence number (29,000). Multivariate statistics on the rhizosphere communities were performed in R (R Development Core Team, 2018). Specifically, the phylogenetic trends of 39 dominant bacterial families (mean recovery ≥0.05% in any sample) from 11 phyla were represented in a heat map with hierarchal clustering using the *heatmap* function in the “gplot” package (Oksanen et al., 2013). Venn diagrams created with the “venneuler” package were used to examine differences between OTUs in the different rhizosphere samples. The Illumina sequence reads are available at the NCBI Sequence Archive under BioProject ID PRJNA553550, BioSample accessions SAMN12238110, SAMN12238111, SAMN12238112, SAMN12238113, SAMN12238114, SAMN12238115, SAMN12238116, SAMN12238117, SAMN12238118, SAMN12238119^3^.

### Analysis of Biofilm Formation of Isolates

Bacterial isolates were tested for the ability to form biofilms in 96 well plates, generally following published protocols (Coffey and Anderson, 2014) with minor modifications. Briefly, overnight liquid cultures were diluted to an OD_600_ of 0.4, and 100 μl was seeded into each well of a 96 well plate. Each culture was seeded in triplicate in random locations in the plate to avoid position effects. The plate was sealed and incubated at 30°C for 24 h (without any shaking). The liquid media was then carefully removed and the wells were stained with 100 μl of 0.01% crystal violet for 20 min at room temperature. The stain was then removed, wells were washed twice with sterile distilled water, and the remaining dye in each well was solubilized by adding 100 μl of 30% acetic acid and pipetting up and down to fully suspend and mix the dye. The plate was scanned at OD_570_ to measure biofilm levels for each sample.

### Plant Growth Stimulation Trials With Microbiome Isolates

Individual isolates were evaluated for the ability to stimulate growth of young alfalfa seedlings when used as an inoculum. These initial trials were done with autoclaved soil and sterilized seeds in closed pots (see details below) to remove any bacteria from the soil and on or within the seeds, to ensure that the only bacteria present would be the inoculum (except for the uninoculated controls). Alfalfa seeds were sterilized with dilute bleach (1% sodium hypochlorite) for 10 min, followed by two washes with sterile water and incubation for 1 h in 70% ethanol, followed by four washes with sterile water (all steps at room temperature). The seeds were then allowed to germinate in a sterile petri dish in a small amount of water. After 36–48 h, the seedlings were transplanted into autoclaved soil (1:1:1 Miracle Grow potting soil^4^:clay:sand) in a clear magenta box. One hundred ml of 0.5 X Hoagland’s basic nutrient solution containing 0, 0.5, or 1% NaCl (or as indicated if otherwise) along with 1 ml of the bacterial culture to be tested as inoculum was added to each box. *Bacillus* strain GB03 was obtained from the Bacillus Genetics Stock Center (bgsc.org, stock ID 3A37) and also tested for growth promotion of alfalfa in the presence of salt. Similar samples without bacteria (sterile LB broth only) were included as experimental controls. Three seedlings were transplanted into each box, repeated for a total of six replicates (two boxes per inoculum or control for a total of 6 plants per treatment). For each replicate box a second magenta box was inverted and taped in place with a small gap (~2 mm) on one side to allow for air exchange while reducing evaporation. Boxes were placed in a plant growth room with a 16 h light (82 μmol m^−2^ s^−1^)/8 h dark cycle at 22°C and ambient humidity with no further watering. After 6 weeks of growth, plant height and total weight and length of shoots and roots were measured. Uninoculated plants were included as controls. After confirming normality of the data, differences in shoot and root length among inoculated and control plants were determined using one-way ANOVA with a Tukey’s HSD test using R.

To confirm the presence of the bacterial inoculum at the conclusion of the growth experiment, soil and root samples were collected when the plants were harvested. Soil was diluted in sterile PBS and spread on LB agar plates containing 1 M NaCl as before. Roots were surface sterilized, ground in sterile PBS, and similarly spread on plates. DNA was isolated from colonies and sequenced as before, and colony morphology was compared to confirm that the recovered bacteria were the same as those used to inoculate the plants.

### Greenhouse Trials

The next step was to test the bacterial isolates in open pots in the greenhouse. For this, alfalfa seeds were surface-sterilized with 50% Chlorox^®^ bleach for 10 min, rinsed with sterile water 5 times, and germinated in an incubator for 2 days. Three seedlings were transplanted into open pots (15 cm round) containing Miracle-Gro^®^ Potting mix (See text footnote 4) and grown in the greenhouse under natural light with temperatures at 25 ± 2.0°C/day time and at 18 ± 2.0°C/night time, and humidity with 45–70%. On the following day, each seedling was inoculated with 1 ml of halophilic bacteria at 1.0 of OD_600_ suspended in PBS buffer. Control uninoculated seedlings were supplemented with 1 ml of PBS buffer. Each treatment had 10 pots. Salt treatment started 7 days after halophilic bacterial inoculation with 1% NaCl solution. Plants were harvested 1 month after salt treatment. Soil was washed out with tap water, and lengths and fresh weights of shoots and roots were measured. Data analysis was conducted with one-way ANOVA and LSD comparison using SAS University Edition.

## Results

### Recovery and Characterization of Rhizospheric and Endophytic Bacteria

The collection site primarily consists of highly saline soil with three dominant halophyte species, *Allenrolfea occidentalis, Salicornia rubra,* and *Sarcornia utahensis* (Figure 1). This site is just south of Utah Lake with high salinity due to the evaporation of water since the collapse of ancient Lake Bonneville more than 14,000 years ago (Weber, 2016). This area is about 1.5 miles away from productive alfalfa fields where soil is much less saline (0.7–1.6 dS/m compared to 16–100 dS/m where the halophyte samples were collected). Soil salinity around the plants ranged from 16 to 18 dS/m in the spring, and up to 70 dS/m in the fall (Table 1). This variation is likely due to the majority of rainfall occurring during the winter and early spring months followed by very dry summers. In areas where no plants were growing salinity was between 45 and 100 dS/m depending on the season. All soil samples had a pH between 7.56 and 7.98 (Table 1).

**Table 1:** Physicochemical analysis of soil samples.

	Spring (April) 2018		Fall (October) 2018	
Plant species	EC dS/m	pH	EC dS/m	pH
*Allenrolfea* and *Sarcornia*	16	7.56	70	7.8
*Salicornia rubra*	18	7.74	70	7.8
Bare-no plants	45	7.98	100	7.7

Bacterial isolates were recovered from the rhizosphere samples on LB agar plates containing 1 M NaCl. Isolates were found to have varying levels of maximum salt concentration tolerance for growth, with some growing in the presence of up to 4 M NaCl (Table 2). The isolates grew equally well on minimal media agar plates at the same salt concentrations. The temperature range for growth, pigmentation, and colony morphology were recorded for each isolate (Table 2). Colony morphology aided in identification of genus (Vreeland et al., 1980; Zhang et al., 2007). For example, *Kushneria* forms bright red-orange colonies (Sanchez-Porro et al., 2009).

**Table 2:** Identification of some microbiome species associated with the halophytes.

Genus/species and accession no.	Family	Order	Phylum	Max. salt tolerance	Temp. range °C	Colony pigment/morphology	Biofilm formation	Gram stain/cell morphology
*Zhihengiluella halotolerans* MK873900	Micrococcaceae	Actinomycetales	Actinobacteria	3 M	N/A	Shiny yellow	**	Gram + very short rods
*Halomonas elongata* MK873884	Halomonadaceae	Oceanospirillales	Gamma- Proteobacteria	4 M	22–42	White, shiny	***	Gram – short rods
*Bacillus sp.* MK873882	Bacillaceae	Bacillales	Firmicutes	3 M	N/A	Dull orange	*	Gram + short fat rods
*Virgibacillus sp.* MK873894	Bacillaceae	Bacillales	Firmicutes	3 M	N/A	White	N/A	Gram + rods
*Kushneria marisflavi* MK873879	Halomonadaceae	Oceanospirillales	Gamma- Proteobacteria	3 M	N/A	Red-orange, shiny	***	Gram – short stubby rods
*Halomonas huangheensis* MK873906	Halomonadaceae	Oceanospirillales	Gamma- Proteobacteria	3 M	22–42	Brown large, shiny	**	Gram – rods, very short, nearly oval
*Bacillus licheniformis* MK873893	Bacillaceae	Bacillales	Firmicutes	3 M	22–42	White round, flat	–	Gram + long rods
*Bacillus sonorensis* MK873902	Bacillaceae	Bacillales	Firmicutes	1.5 M	22–42	Dull yellow small, round	–	Gram + long filamentous rods

GenBank accession numbers are provided below the most probable genus and species name of each isolate. Biofilm formation is characterized as: –, no detectable biofilm; *, detectable but low level of biofilm; **, moderate biofilm formation; ***, strong biofilm formation.

### DNA Sequence Analysis and Bacterial Species Identification

BLAST analysis of the 16S rRNA amplicon sequences from 41 independent isolates was performed to identify the bacteria recovered (details are available for each *via* the GenBank accession numbers that are included in Materials and Methods for all isolates and in Table 2 for selected isolates). Many of the isolates were identified from the same genus and could not be further identified at the species level based on colony morphology or Gram stain. The most common bacterial genera recovered were *Halomonas* (16 of the 41 isolates tested)*, Bacillus* (16 isolates), and *Virgibacillus* (4 isolates). There were two isolates from *Kushneria* and one isolate each from *Oceanobacillus, Vibrio,* and *Zhihengiluella*.

**Figure 2:**
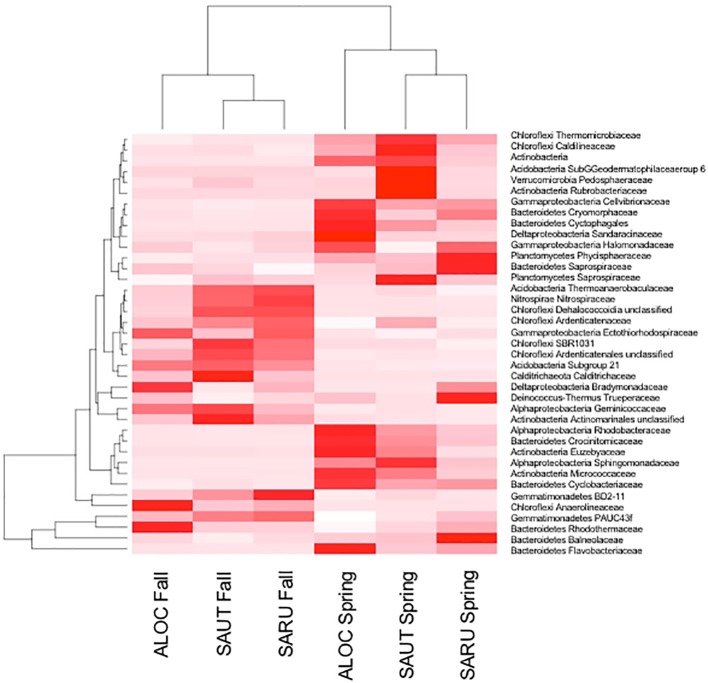
Rhizosphere bacteria of the three halophytes in spring and fall samplings. Heat map and dendrogram showing relationships between the abundance of major bacterial families and samples collected in the fall and in the spring. ALOC, *Allenrolfea occidentalis*; SAUT, *Sarcocornia utahensis*; SARU, *Salicornia rubra*.

**Figure 3:**
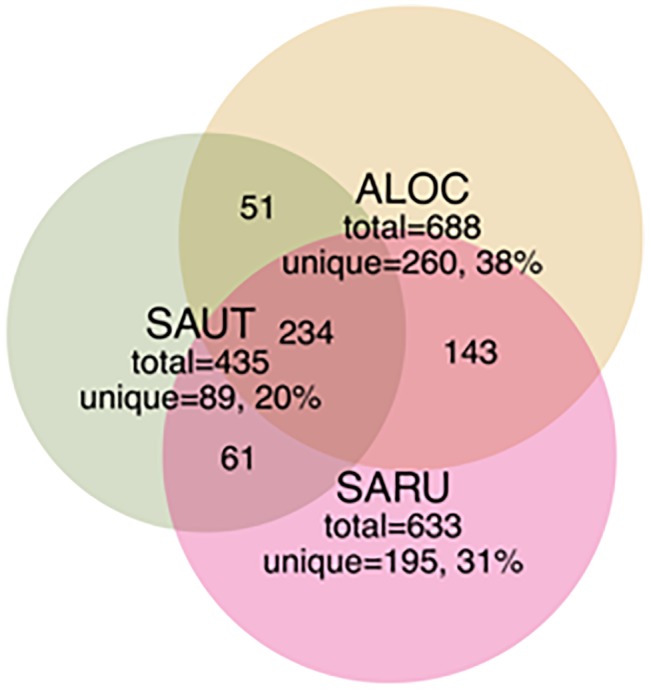
Venn diagrams showing the distribution of shared and unique rhizobacterial species between the three halophyte species. Recovery was based on OTUs from bacterial community libraries of the 16S rRNA gene (97% similarity cutoff), with numbers indicated in each quadrant (not to scale). Abbreviations are as in Figure 2.

To obtain a more detailed picture of total bacterial diversity associated with each halophyte, total rhizosphere DNA was analyzed by Illumina sequencing. Next-generation sequencing of the 16S rRNA gene (shown in Figures 2, 3) identified some similar OTUs as the plated isolates. For example, *Halomonas, Kuchneria, Bacillus* and several others were identified by both approaches. Further, bacterial communities were sensitive to seasonal fluctuation from the spring to fall (Figure 2). This is likely at least partially due to the significant difference in soil salinity, increasing from 16 to 18 dS/m in the spring to about 70 dS/m in the fall when samples were collected from the halophytes, while soil pH remained about the same. Based on unique OTUs in rhizospheres, bacterial communities were more unique on roots of *Allenrolfea occidentalis* than *Salicornia rubra* and *Sarcocornia utahensis* (Figure 3). For example, the number of unique OTUs in *Allenrolfea occidentalis* rhizospheres was at least 1.3-times higher than the *Salicornia* species.

Species or OTUs identified were from the Cryomorphaceae, Cytoophagales, Flavobacteriaceae, Rhodothermaceae (Bacteriodetes), and Anaerolineaceae (Chloroflexi). Bacterial community results were based on the recovery of 175,239 quality sequences and 3,550 unique OTUs with samples possessing an average sequencing coverage of 97% ±0.003 (mean ± SEM).

### Characterization of Isolates for Biofilm Formation

The *Halomonas, Kushneria, and Zhihengliella* isolates form biofilms when grown in LB + 0.25 M NaCl, while the other isolates tested do not form or poorly form detectable biofilm (summarized in Table 2). Biofilm formation by some bacterial strains has been shown to be associated with soil adherence to plant roots in some studies (Qurashi and Sabri, 2012).

### Screening of Isolates for Alfalfa Growth Stimulation Capabilities

**Figure 4:**
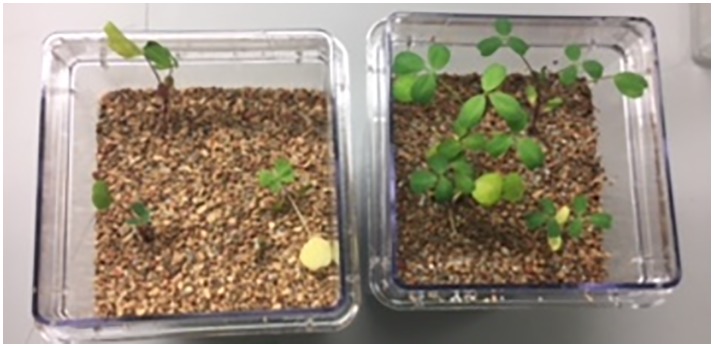
Growth stimulation of alfalfa seedlings in soil in the presence of 1% salt. Uninoculated control (LB media without bacteria), left. Inoculation with the Bacillus isolate, right.

**Figure 5:**
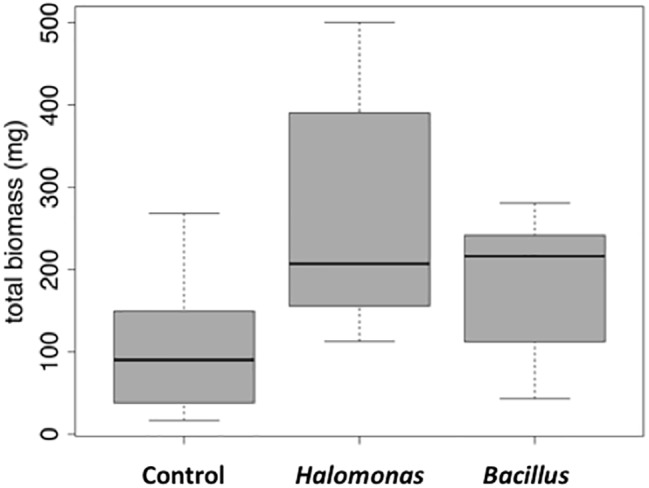
Box and whisker plot of stimulation of alfalfa growth by bacterial inoculation in the presence of 1% NaCl. Total mass is in milligrams. LB, control (no bacterial inoculation). Grown in the laboratory in replicate pots with three plants per pot.

We next tested the salt-tolerant bacterial isolates for the ability to stimulate growth of alfalfa under saline conditions. This screening identified *Halomonas* (MK873884) and *Bacillus* (MK873882) isolates that significantly stimulated growth when used to inoculate alfalfa (Figures 4, 5). Total biomass was 2.4-times higher in alfalfa inoculated with *Halomonas* than uninoculated alfalfa (one-way ANOVA, *F* = 3.1, *p* = 0.06, *df* = 2). While this has only borderline significance, similar results were obtained with repeated trials. Some other isolates appeared to inhibit or to have little effect on plant growth. A few strains had a slight stimulatory effect on plant growth, including some *Pseudomonas* species*, Kushneria*, *Bacillus subtilis* strain GB03, *Bacillus licheniformis* and some mixed cultures (not shown).

### Recovery of Inoculum From Soil and Roots of Inoculated Plants

To determine whether the bacterial inoculum was able to colonize the soil and/or become endophytic in alfalfa roots, soil and root samples were collected when the alfalfa plants were harvested and plated as before. Colonies showed the same characteristics as the bacteria used to inoculate the plants, and DNA was isolated and sequenced to confirm identity [*Halomonas* (MK873884) and *Bacillus* (MK873882)]. Roots from plants inoculated with these two isolates also yielded the same bacteria (ranging from 3,000 to 8,000 colonies per gram of soil) used to inoculate the plants, while the control LB plants and those inoculated with one of the other *Bacillus* isolates did not yield bacteria. The observation that the *Halomonas* and *Bacillus* isolates were able to form endophytic relationships with alfalfa leading to growth stimulation is encouraging for their potential use as inoculants to enhance growth of non-host plants under saline conditions.

### Growth Stimulation in Greenhouse Studies

**Figure 6:**
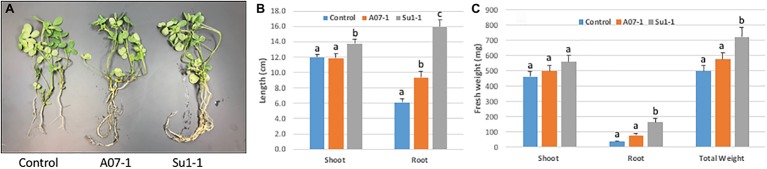
Alfalfa growth stimulation by halophilic bacteria in salty soil. **(A)**. The photo shows 3 representative plants from each treatment. **(B)**. Significant root length increase induced by the *Halomonas* (A07–1) and *Bacillus* (Su1–1) isolates. **(C)**. Plant growth performance enhanced by halophilic bacteria. Each treatment had 30 plants, and plants were watered with 1% NaCl solution starting 1 week after bacterial inoculation and grown in the greenhouse.

The initial growth stimulation trials were performed in small closed pots in a controlled environment. We next wanted to scale up the experiments in greenhouse trials at the Institute for Advanced Learning and Research. Alfalfa plants were grown in open pots with carefully controlled watering and growth monitoring. As with the earlier studies, plants were grown with and without inoculation with the *Halomonas* and *Bacillus* isolates, in the presence and absence of 1% NaCl in the watering solution. In the absence of salt in the watering solution there were no differences in either shoot or root biomass between halophilic bacterial inoculation and control treatment. The inoculation of both *Halomonas* and *Bacillus* isolates stimulated alfalfa root growth (Figure 6), with root length increasing 2.6-fold in *Halomonas* and 1.5-fold in *Bacillus* inoculated plants relative to uninoculated control alfalfa (one-way ANOVA, *F* = 43.85, *p* < 0.0001, df = 2). Shoot length was also elevated but only for *Bacillus* (one-way ANOVA, *F* = 3.23, *p* = 0.0444, df = 2). In addition, *Bacillus (Su1–1)* showed much better performance than *Halomonas (A07–1)* in root and shoot biomass, with at least 4.5-fold increase in root fresh weight over the control treatment (one-way ANOVA, *F* = 14.45, *p* < 0.0001, df = 2) and only 21% increase in shoot fresh weight over the control treatment (one-way ANOVA, *F* = 1.28, *p* > 0.2848, df = 2). Total fresh weight was significantly increased by *Bacillus (Su1–1)* (one-way ANOVA, *F* = 4.92, *p* < 0.0095, df = 2). In addition, both the *Halomonas* inoculation and uninoculated control treatments had two dead plants while the *Bacillus* inoculation treatment had no dead plants.

## Discussion

Production of sufficient food for the world’s population is a critical challenge, exacerbated by the loss of agricultural land to urbanization, degradation of existing land, diminished water quality, and salinization of soil in many areas. These factors leave farmers in many parts of the world with access only to poor land (low soil quality) and/or poor water quality to produce crops for human consumption and for animal feed. The development of crop plants that are able to adapt and grow sustainably under changing environmental stresses is of urgent importance.

Our objective in this study was to make a general survey of the types of bacteria that are present in association with three species of halophytes in central Utah (*Salicornia rubra, Sarcocornia utahensis*, and *Allenrolfea occidentalis*). We have identified 41 isolates, including multiples from the same genus, of culturable halophilic bacteria. These strains vary in their ability to form biofilms, in the maximum concentration of salt that allows growth, and in pigmentation and colony morphology. Several of the isolates had strong yellow, orange or red pigmentation due to carotenoids that may help protect the bacteria from damaging UV radiation (Khaneja et al., 2010). *Halomonas* species (based on sequencing and colony morphology they are most likely *H. elongata* or *H. huangheensis*) were found as root endophytes and in the rhizosphere of all three halophytes. *Halomonas* and *Kushneria* are closely related, and in the past were grouped in the same genus (Sanchez-Porro et al., 2009). Analysis of total soil or root tissue identified many other non-culturable bacteria, including members of common soil phyla and some that are present in extreme environments such as desert and saline conditions. The rhizosphere of *Allenrolfea occidentalis* supported the highest number of unique OTUs (260 OTUs or 38% of OTUs), while *Sarcocornia utahensis* supported the lowest number of unique species (89 OTUs or 20% of OTUs). At least 34% of rhizosphere OTUs were shared among the three species.

A very important advance resulting from the screening of isolates for plant growth promotion capabilities was the identification of two that support growth of alfalfa in saline soil when used to inoculate young seedlings. When used to inoculate alfalfa seedlings, *Halomonas* and *Bacillus* stimulated alfalfa growth in soil watered with 1% NaCl, with *Bacillus* showing the greater stimulation of growth of both shoots and roots. Bacteria recovered from roots of inoculated alfalfa were the same as used for the inoculation, suggesting that these strains may be useful for inoculation of alfalfa to enhance plant growth in salty soil.

Several other isolates tested did not stimulate growth of alfalfa in the presence of salt. We tested the *Bacillus* GB03 strain that previously was shown to stimulate plant growth in the presence of salt (Xie et al., 2009; Han et al., 2014), but did not observe noticeable growth stimulation of alfalfa in saline soil. This suggests that the GB03 strain may enhance growth of only some plant species in the presence of salt. It is likely that different mechanisms are involved in each bacterial/plant interaction that leads to plant growth stimulation under saline conditions, and that a bacterial strain that stimulates growth of one plant species in the presence of salt may not cause similar stimulation in other plants. Some examples include ginseng, where *Paenibacillus yonginensis* strain DCY84^T^ protects against salinity stress by induction of defense related systems including ion transport, ROS enzyme production, proline content, total sugar and ABA biosynthesis related genes (Sukweenadhi et al., 2018). Another research group found that an endophytic strain of *Bacilllus amyloliquefaciens* produces ABA in response to increasing salinity, increasing production of glutamic acid and proline to increase resistance to salinity in rice (Shahzad et al., 2017). In addition to these examples, there are multiple reports of different bacterial species that stimulate growth of a variety of plant species, supporting the notion that stimulation may be specific to the plant host and bacterial species (Mitter et al., 2013; Bharti et al., 2016; Li et al., 2016; Navarro-Torre et al., 2016; Yuan et al., 2016; also see Introduction).

The mechanisms by which halophilic bacteria stimulate plant growth include binding of salt ions by the bacteria or production of volatile compounds or other signals that stimulate expression of genes to enhance growth *via* increased photosynthesis or other changes in the host plant (Meena et al., 2017; Numan et al., 2018). Some microbes produce biofilms in the rhizosphere that trap water and nutrients and reduce plant uptake of sodium ions from the soil (Nadeem et al., 2014).

There are several mechanisms that may be involved in plant growth promotion by endophytes under non-saline conditions (Kim et al., 2012; Santoyo et al., 2016). Mechanisms by which endophytes enhance plant growth include acquisition of nutrients and altering expression of plant genes that affect growth and development. The endophyte *Burkholderia phytofirmans* PsJN enhances growth for six of the eight switchgrass cultivars that were tested (Kim et al., 2012). Inoculation with this strain was found to induce wide-spread changes in gene expression in the plant host, including transcription factors that are known to regulate expression of some plant stress factor genes (Lara-Chavez et al., 2015). It is likely that changes in plant gene expression are also induced by the halophilic bacteria used in this study to inoculate alfalfa. This is a focus of current research in the laboratory.

In summary, in this study we have produced a general survey of bacterial species associated with native Utah halophytes growing in highly saline soil. We have partially characterized 41 isolates that can be cultured in the laboratory and those that are not culturable but can be identified from DNA isolated from soil. Both approaches led to identification of bacteria from the same genera. We subsequently identified and characterized plant growth promotion activity of two of these isolates (*Halomonas* and *Bacillus*). When either of these isolates was used to inoculate alfalfa, significant enhancement of plant growth in the presence of salt was observed. The promising results from this study warrant further, in-depth analysis of plant growth promotion by these and other halophilic bacterial species. This research will have a significant impact on efforts to identify bacteria that stimulate growth of other plant species under a variety of stress conditions.

## Author Contributions

BN generated ideas, obtained funding, directed the work, conducted some experiments, wrote and edited manuscript. JK, CMc, and SS isolated and characterized initial collection of bacterial strains including sequence analysis. JW, EC, and MH conducted laboratory growth promotion studies and analyzed data. JL and CMe performed the greenhouse studies and resulting data analysis for Figure 6. ZTA conducted the metagenomics analysis and prepared some figures. All coauthors reviewed the manuscript.

### Conflict of Interest Statement

The authors declare that the research was conducted in the absence of any commercial or financial relationships that could be construed as a potential conflict of interest.

## Footnotes

^1^https://www.ars.usda.gov/pacific-west-area/riverside-ca/us-salinity-laboratory/

^2^http://dnasc.byu.edu/

^3^https://www.ncbi.nlm.nih.gov/Traces/study/?acc=PRJNA553550

^4^miraclegro.com
